# The advent of chimeric antigen receptor T Cell therapy in recalibrating immune balance for rheumatic autoimmune disease treatment

**DOI:** 10.3389/fphar.2024.1502298

**Published:** 2024-12-12

**Authors:** Qianyu Guo, Jie Li, Juanjuan Wang, Linxin Li, Jia Wei, Liyun Zhang

**Affiliations:** ^1^ Department of Rheumatology, Shanxi Bethune Hospital, Shanxi Academy of Medical Sciences, Third Hospital of Shanxi Medical University, Tongji Shanxi Hospital, Taiyuan, China; ^2^ Third Hospital of Shanxi Medical University, Shanxi Bethune Hospital, Shanxi Academy of Medical Sciences, Tongji Shanxi Hospital, Taiyuan, China; ^3^ Department of Hematology, Shanxi Bethune Hospital, Shanxi Academy of Medical Sciences, Tongji Shanxi Hospital, Third Hospital of Shanxi Medical University, Taiyuan, China; ^4^ Sino-German Joint Oncological Research Laboratory, Shanxi Bethune Hospital, Shanxi Academy of Medical Sciences, Taiyuan, China; ^5^ Department of Hematology, Tongji Hospital, Tongji Medical College, Huazhong University of Science and Technology, Wuhan, China; ^6^ Immunotherapy Research Center for Hematologic Diseases of Hubei Province, Tongji Hospital, Tongji Medical College, Huazhong University of Science and Technology, Wuhan, China

**Keywords:** CAR-T cell, autoimmune disease, systemic lupus erythematosus, rheumatoid arthritis, Sjögren’s syndrome, systemic sclerosis, idiopathic inflammatory myopathies, ANCA-associated vasculitis

## Abstract

CAR-T cell therapy, a cutting-edge cellular immunotherapy with demonstrated efficacy in treating hematologic malignancies, also exhibits significant promise for addressing autoimmune diseases. This innovative therapeutic approach holds promise for achieving long-term remission in autoimmune diseases, potentially offering significant benefits to affected patients. Current targets under investigation for the treatment of these conditions include CD19, CD20, and BCMA, among others. However, CAR-T therapy faces difficulties such as time-consuming cell manufacturing, complex and expensive process, and the possibility of severe adverse reactions complicating the treatment, etc. This article examines CAR-T therapy across various rheumatic autoimmune diseases, including systemic lupus erythematosus (SLE), rheumatoid arthritis (RA), Sjögren’s syndrome (SS), systemic sclerosis (SSc), antisynthetase syndrome (ASS), and ANCA-associated vasculitis (AAV), highlighting both therapeutic advancements and ongoing challenges.

## 1 Introduction

Autoimmune diseases are typically chronic conditions characterized by a wide range of clinical manifestations affecting multiple organs (Ameer et al., 2022; Radu and Bungau, 2021; Manfrè et al., 2022; Bongartz et al., 2007; Adami et al., 2019). The pathogenic mechanisms underlying these diseases remain largely unresolved, and they are often incurable. These conditions frequently result in high rates of disability and mortality. The *status quo* of high medical costs associated with long-term medication use, in addition to the fact that some patients are ineffective or intolerant to conventional medications (antirheumatic drugs, nonsteroidal anti-inflammatory drugs, and glucocorticosteroids) (Lin et al., 2020; Huang et al., 2021), makes the treatment of autoimmune diseases an important clinical issue.

It is now well established that B cells play a crucial role in various autoimmune diseases. Several commonly used biologics exert their therapeutic effects by targeting and inhibiting B cell activation or proliferation. For instance, rituximab, an anti-CD20 agent frequently used for B cell-mediated diseases (Lee et al., 2020), is costly, necessitates frequent injections, and does not fully deplete B cells to achieve complete remission (Cataldi et al., 2017). Thus, there is a clear need to investigate alternative treatment modalities.

CAR-T cell therapy has demonstrated remarkable efficacy in treating leukemia and malignant tumors. Leveraging its therapeutic principle—where CAR-T cells are engineered to target and eliminate pathological immune cells—offers promising insights for its application in autoimmune diseases (Kansal et al., 2019; Jin et al., 2021). Several clinical studies of CAR-T for the treatment of autoimmunity have been conducted with encouraging results (Qin et al., 2023; Lundberg et al., 2023). This article provides an overview of the advancements in utilizing CAR-T therapy for treating autoimmune diseases. Our center has also conducted a clinical trial registered with the NCT for patients with relapsed and refractory systemic lupus erythematosus (SLE), where CAR-T therapy has demonstrated promising efficacy in treating autoimmune conditions.

## 2 Mechanisms underlying CAR-T therapy in autoimmune diseases

**FIGURE 1:**
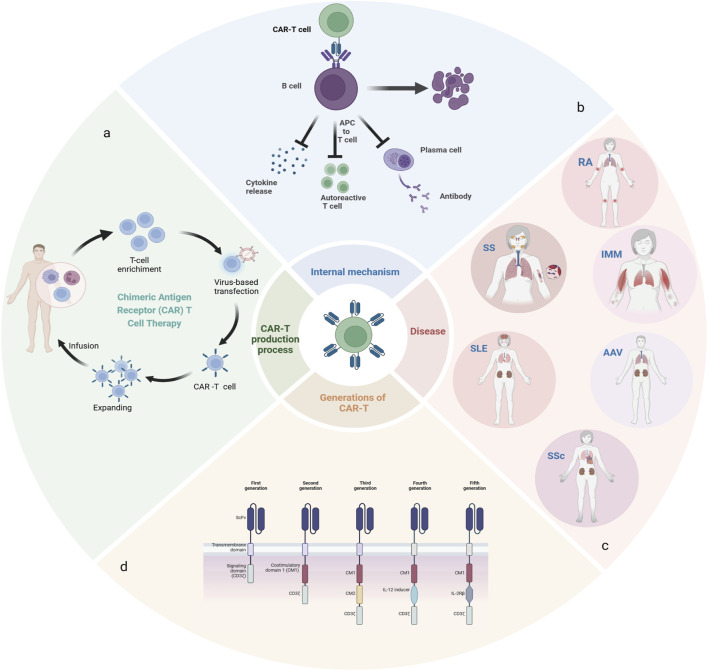
**(A)**: The manufacturing process of CAR-T; **(B)**: The mechanism of action of CAR-T *in vivo*; **(C)**: Rheumatic autoimmune diseases related to abnormal B cell function; **(D)**:5 generations of CAR-T therapy development.

Pathologic cells associated with autoimmune diseases include autoantibody-producing B cells, activated T cells, and antigen-presenting cells (antigen-presenting cells, APCs) (Boulch et al., 2021). Among them, B cells are mainly responsible for the humoral immune response and play an important role in a variety of autoimmune diseases by producing antibodies, releasing pro-inflammatory cytokines and activating T cells as APCs (Oh and Payne, 2022) (Figure 1B).

Systemic lupus erythematosus is a complex disease characterized by antibody production and heterogeneity of clinical manifestations, with disruption of B-cell tolerance being a critical early event in the course of the disease (Krustev et al., 2023; Möckel et al., 2021). Rheumatoid arthritis is a chronic, progressive autoimmune disease in which defects in B-cell tolerance checkpoints, autoreactive B cells acting as antigen-presenting cells (APCs) to stimulate the activation of autoreactive T cells, and the production of proinflammatory and anti-inflammatory cytokines, are functions that, either individually or in combination, contribute to the pathogenesis of RA ((Finnegan et al., 2012)). In addition, multiple B cell subpopulations have been found in the salivary glands of patients with desiccation syndrome (Daridon et al., 2006; Qin et al., 2022; Verstappen et al., 2021; Rivière et al., 2021), and BAFF plays a prominent role in its pathogenesis as an essential survival factor for both transitional and mature B cells (Zhan et al., 2023). Systemic sclerosis is also a systemic autoimmune disease, mainly characterized by autoimmune abnormalities, especially B cells play an important role in the development of systemic sclerosis. Patients with this disease often present with hypergammaglobulinemia and the presence of various autoantibodies (Fukasawa et al., 2024). Anti-synthetase syndrome is a distinct subtype of idiopathic inflammatory myopathy, characterized by the presence of autoantibodies—particularly anti-Jo-1 antibodies—which are frequently associated with pathological manifestations such as interstitial lung disease, Raynaud’s phenomenon, and arthritis. Given these associations, deep depletion of B cells may offer therapeutic benefits. Anti-neutrophil cytoplasmic autoantibody (ANCA)-associated vasculitis (AAV) is a life-threatening systemic autoimmune disease, driven by autoantigens such as myeloperoxidase (MPO) or protease 3. Therapeutic strategies have been explored to achieve clinical benefits by depleting B cells that produce MPO-ANCA((McClure et al., 2018)) (Figure 1C).

Current treatments for autoimmune diseases focus on suppressing the immune system through small molecules that inhibit immune cell activation or proliferation, such as the B-cell depleting drug anti-CD20 monoclonal antibody rituximab, which was initially approved for use in certain types of B-cell malignancies and is now used to treat B-cell mediated autoimmune disease. However, some patients do not show remission, and even when effective, frequent injections are required and there is a risk of chronic B-cell failure and infection due to chronic immunosuppression (Tony et al., 2011). The drug has also been found to struggle with the complete elimination of B cells, as evidenced by the persistence of B cells within the synovium of rheumatoid arthritis patients treated with rituximab. This suggests that tissue-resident B cells may evade depletion by rituximab, in contrast to circulating B cells (Thurlings et al., 2010).

Chimeric antigen receptor T-cell (CAR-T) immunotherapy is a novel therapeutic approach that involves the extraction of T cells, either autologous or allogeneic, followed by *in vitro* expansion and subsequent infusion into the patient (Figure 1A). Genetic material with specific antigen recognition domains and T-cell activation signals are transferred into T cells by retroviruses or lentiviruses, so that T cells no longer require APCs for activation, and these CAR-T cells initially bind to antigen-expressing target cells and subsequently exert their cytotoxic effects, leading to the lysis of the target cells. In addition, CAR-T cells can differentiate into long-lived memory cells *in vivo*, facilitating more sustained remission. Leveraging the cytotoxicity and tissue infiltration capabilities of T cells, CAR-T cells can also effectively target difficult-to-reach, tissue-resident tumor cells. In comparison, conventional drugs are ineffective or intolerable for many patients, and biologic anti-cd20 antibodies require repeated administration to maintain therapy (Reddy et al., 2015). And over time, the host can become resistant to treatment, all of which further limits the use of conventional drugs.

A classic CAR has three main portions: ecto-domain, transmembrane domain and endo-domain. The ecto-domain includes a recognition domain of the antigen and a hinge domain, and the endodomain is characterized as a costimulatory and intracellular signaling domain. The first generation of CARs only consisted of three simple parts, there was no co-stimulation part, which could not secrete enough cytokines and led to the failure of amplification. Therefore, co-stimulation signals were added on the basis of the first generation, and the second generation of CARs was born. The co-stimulation signal can induce the proliferation of T cells and the release of cellular factors. The subsequent generations were designed on the basis of the second generation, the third generation added a stimulating factor on the basis of the second generation, and the fourth and fifth generations added cytokines to regulate the tumor microenvironment to enhance efficacy (Figure 1D) (Chen et al., 2023; Baker et al., 2023; Adabi et al., 2023).

## 3 Classification of CARs

### 3.1 Classification of CARs according to their cell type

CAR-T cells are genetically engineered T cells designed to express synthetic receptors on their surface, enabling them to identify and eliminate cancer cells by recognizing specific tumor antigens independently of human major histocompatibility complex (MHC) molecules (Chen et al., 2023). Initially conceived as a “living drug” to target and destroy tumors, CAR-T cells have shown promising efficacy in this regard. Natural killer (NK) cells eliminate target cells through two primary mechanisms: the release of cytolytic granules and the induction of apoptosis via Fas ligand (FasL). The incorporation of chimeric antigen receptors (CARs) enables both T cells and natural killer (NK) cells to selectively and effectively target and eliminate cancer cells. And it was observed that CAR NK has a better safety profile (Obstfeld et al., 2017). It also paves the way for the development of off-the-shelf CAR products that can be cryopreserved for long-term storage (Siegler et al., 2018). However, CAR-NK cells suffer from limited durability. A significant challenge for both CAR-T and CAR-NK cells in the treatment of solid tumors is their difficulty in effectively infiltrating the tumor’s interior. So researchers began introducing CARs into macrophages, which are able to infiltrate solid tumors naturally and effectively (Morrissey et al., 2018; Sternlicht and Werb, 2001; Klichinsky et al., 2020). To overcome these drawbacks, other cell therapies are being developed, and currently there are NK cells, macrophages, Treg cells, neutrophils, and pluripotent stem cell-derived CARs cell therapies (Table 1).

**TABLE 1:** Classification of CARs according to different classification methods.

Classification	Type
Cell types of CAR	T cells, NK cells, Macrophages, Treg cells, Neutrophils, Pluripotent stem cells
Target	CD19、BCMA、CD19+CD22、CD22、CD7、CD9+CD20、CD123、CD20、CD30、BCMA + CD19、CD33、CD5、CD138、NKG2D、CD4、BCMA + TACI、BAFF、CD19+CD20^+^CD22、BCMA + GPRC5D、GPRC5D 、SLAMF7、CD44v6、TRBC1、FLT3、CD19+CD70、CD19+CD79b、CD20+CD22、CD38、Siglec-6、CD33+CLL1、IgBeta、CD20+CD 79b、Mesothelin、GPC3、MUC1、GD2、HER-2、NKG2D、EGFR、B7-H3、Claudin、CEA、PSMA、CD70、ROR1、FAP、T4、GD2/CD70、GD2/CD56、GD2/PSMA、CD207、MMP2、BT-001、GUCY2C、TAG72 [(Wang et al., 2023)]
Sources of CAR-T and Infusion Methods	Autologous CAR-T、Allogeneic CAR-T、Engineering CAR-T *in vivo*

### 3.2 Classification according to target

CD19 is a surface antigen predominantly expressed on B-lineage malignancies. It has become a prominent target for CAR-T therapy, beginning with the approval of Kymriah as the first anti-CD19 CAR product in 2017. Since then, CD19 CAR-T therapy has demonstrated success in clinical trials across various B-cell cancers (Wei et al., 2019; Kochenderfer et al., 2015). And gradually more targets such as BCMA, CD22, CD7, CD20, etc., have also been introduced in order to deeply deplete B cells (Table 1).

### 3.3 Classification according to the source of CAR-T and the mode of infusion

The manufacturing process for autologous CAR-T cells takes approximately 3 weeks, during which patients may experience disease progression. To address this issue, universal CAR-T cells derived from allogeneic sources have been proposed as an alternative. It is expected to be produced on a large scale as an “off-the-shelf” drug. Recently, Wang et al. reported the first use of allogeneic CAR-T cells in patients with autoimmune diseases. All three patients showed significant clinical improvement. And they showed significant clinical improvement and remained in remission for the 6 months of follow-up to date (Wang X. et al., 2024; Baker and June 2024). A major adverse event of allogeneic CAR-T therapy is the risk of GvHD due to attack of host organs by the injected T cells. No signs of GvHD have been reported in these patients, and another important safety issue in CAR-T therapy is CRS. However, Wang et al. did not observe any clinical signs of severe CRS. This may be due to the fact that the target burden of autoimmune diseases is much smaller compared to cancer. Alternatively, new approaches are being explored to eliminate *in vitro* manufacturing time through gene editing, namely *in vivo* cell reprogramming for *in situ* cell therapy (Nicolai et al., 2024). Currently, *in situ* reprogramming of target cells includes both viral and non-viral approaches. Viral vectors can mediate effective delivery, but are accompanied by immunogenicity and safety issues. Synthetic nanomaterials, such as lipid nanoparticles (LNPs), which have smaller immunogenicity, are easy to produce and low-cost, are promising alternatives to viral vectors, although they are still in the initial stages of exploration (Table 1).

## 4 Advances in CAR⁃T therapy for autoimmune diseases

### 4.1 CAR-T for systemic lupus erythematosus

Systemic lupus erythematosus (SLE) is a highly heterogeneous autoimmune disease that affects almost all organs and tissues (Tsokos et al., 2016). Disruption of immune tolerance and persistent autoantibody production are the two main features of SLE. Current CAR-T cell therapy targets for the treatment of systemic lupus erythematosus (SLE) include CD19, CD20, and BCMA. Administering anti-CD19 CAR-T cells in a mouse model of severe lupus nephritis led to improved disease outcomes and prolonged lifespan, indicating the efficacy of this therapy in SLE and underscoring its potential for clinical application (Jin et al., 2021). Elevated numbers of follicular helper CD4^+^ T cells (TFH) have been observed in patients with SLE, particularly during disease flares, with TFH counts correlating with levels of programmed cell death protein 1 (PD-1) expression (Crotty, 2011). Seth D. Reighard et al. designed and evaluated an innovative CAR-NK cell that targets PD-1-expressing cells to eliminate TFH. In short-term *in vitro* co-cultures, these PD-1-targeting CAR-NK cells selectively cleared TFH cells. These results bring new ideas for the treatment of SLE ((Reighard et al., 2020)). CAR Treg cells theoretically hold promise for restoring Treg numbers and function as a treatment for SLE. However, IL-21 secreted by CD4^+^ T cells compromises Treg survival and function, and a highly pro-inflammatory environment can drive Treg cells to convert into IL-17-producing or other inflammatory factor-secreting cells. These challenges underscore the complexities of using CAR Treg cells in SLE treatment, necessitating further investigation (Kim et al., 2015).

So far, 25cases of SLE treated with CAR-T have been reported, including 10cases in Germany (Krickau et al., 2024), 10 cases in China, and five cases in the US [(Boulougoura et al., 2023)]. In August 2021, Georgschett’s team reported the first patient treated with CAR-T for systemic lupus erythematosus, in which dsDNA autoantibody titers and C3 and C4 complement levels returned to normal after 5 weeks of CAR-T cell injections, proteinuria was virtually eradicated, and the Disease Activity Index was reduced from a score of 16 to 0, with no significant side effects (Mougiakakos et al., 2021). The team later reported on nine additional lupus patients treated with CAR-T, all of whom improved significantly (Mackensen et al., 2022; Subklewe et al., 1990; Li et al., 2024; Krickau et al., 2024).

Our center has also conducted a clinical study of CD19 CAR-T for the treatment of systemic lupus erythematosus (NCT06056921), which has been administered to two patients, both of whom have demonstrated improvement and a favorable safety profile.

### 4.2 CAR-T for Sjögre’s syndrome

Sjögre’s syndrome (SS) is a chronic autoimmune disease with multiple manifestations, the main manifestations of which include dryness of the mouth and eyes, as well as systemic complications (Zhan et al., 2023). B-cell overactivity is evidenced by the presence of various autoantibodies, including RF and anti-SSA/SSB antibodies, as well as by hypergammaglobulinemia. Additionally, some patients with systemic sclerosis may develop malignant B-cell lymphomas. B-cell activating factor (BAFF) is a key driver of systemic sclerosis (SS) development, playing a crucial role in promoting B-cell survival and hyperactivity (Nocturne and Mariette, 2018). Certain targeted therapies, such as rituximab, exert their therapeutic effects by depleting B cells. Anti-CD19 CAR-T cell therapy is similar in principle to CD20 monoclonal antibody therapy, which also treats SS through B-cell depletion. However, it has been observed that long-lived plasma cells in many SS patients do not express or express very low levels of CD20, and thus CD20 monoclonal antibodies cannot target them. In contrast to CD20, which is expressed primarily on mature B cells, CD19 is widely expressed throughout the maturation process of pre-B cells into plasma cells. This broader expression profile suggests that CD19 may be a more effective target for immunotherapy. Currently, two Phase I studies (NCT05085431 and NCT05859997) are investigating the safety and efficacy of CD19/BCMA CAR-T cells for the treatment of systemic sclerosis. The results of these studies have yet to be reported. To date, only a single case has been reported from Germany, involving a patient with a 10-year history of dry syndrome and secondary interstitial lung disease, who was subsequently diagnosed with diffuse large B-cell lymphoma (Sheng et al., 2023). Following treatment with anti-CD19 CAR-T cells, the patient achieved complete remission (CR). Notably, by day 90 post-therapy, the patient tested negative for antinuclear antibodies and anti-Ro-52 for the first time. Early in the treatment, the patient exhibited grade 2 cytokine release syndrome and grade 1 neurotoxicity, but the adverse effects were completely controlled after aggressive treatment, proving that they were tolerated reasonably well. As for more long-term efficacy, long-term follow-up is needed to confirm this.

### 4.3 CAR-T for systemic sclerosis

Systemic sclerosis (SSc) is a connective tissue disease marked by autoimmunity, vasculopathy, excessive extracellular matrix deposition, and fibrosis, leading to atrophy of the skin, subcutaneous tissues, muscles, and internal organs, including the digestive system, lungs, heart, kidneys, and central nervous system (Yoshizaki, 2018). Multiple lines of evidence suggest that B cells play a significant role in the pathophysiology of severe systemic sclerosis. And CD19 was found to be significantly increased in expression in memory B cells and naive B cells in SSc (Beesley et al., 2022), so the current target of CAR-T clinical trials for the treatment of SSc is CD19.

The efficacy of the CD20 antibody-targeted drug rituximab (RTX) in the treatment of SSc remains controversial, with the use of rituximab demonstrating significant skin improvement in one randomized controlled trial, but it led to a decrease in forceful lung capacity (FVC) in another study (Ebata et al., 2021).

Changes in Treg populations were also observed in SSc patients, with a disruption of the balance between Treg and Th17 cells, an immune imbalance that has been implicated as a cause of the disease (Fenoglio et al., 2011; Frantz et al., 2018). As potential treatments for systemic sclerosis (SSc), therapies targeting Treg or Th17 cells are being explored with the aim of restoring the balance between these cell types (Liu et al., 2021). In systemic sclerosis (SSc), myelin basic protein (MBP) and myelin oligodendrocyte glycoprotein (MOG) can act as potential targets within the central nervous system (CNS). Human Tregs have been designed to express the Ob2F3 TCR V region to target myelin basic protein (MBP), and experiments suggest that these ob2f3-expressing Treg cells ameliorate experimental autoimmune encephalitis in transgenic mice (Ferreira et al., 2019). These significant findings indicate that engineered CAR-Tregs targeting the CNS have the potential to be developed for cellular therapy in patients with systemic sclerosis (SSc).

Reports have documented six cases of systemic sclerosis treated with CAR-T therapy, all originating from Germany (Wang X. et al., 2024; Bergmann et al., 2023; Müller et al., 2024; Subklewe et al., 1990). After CAR-T cell infusion, none of them experienced serious adverse effects, only mild cytokine release syndrome (CRS) grade 1, no signs of neurotoxicity, and all of them demonstrated autoantibody turnaround and rapid improvement of multisystemic manifestations of cardiac, joint, and cutaneous involvement in subsequent short-term follow-up, these data offer the initial evidence that targeted CD19 CAR-T cell therapy may be effective in treating severe cases of systemic sclerosis (Feng et al., 2022), but they do not fully clarify whether it is only a short-term effect of CAR-T cell therapy, and further observation of the extent of drug-free remission in SSc is warranted.

### 4.4 CAR-T for idiopathic inflammatory myopathies

Idiopathic inflammatory myopathies (IIM) are a heterogeneous group of autoimmune disorders. Muscle weakness is usually the typical clinical presentation, but other organs may also be affected, including the skin, joints, lungs, heart, etc. Different myositis-specific auto-antibodies have been identified and IIM can be classified into several subgroups - dermatomyositis (including amyopathic dermatomyositis), antisynthetase syndrome, immune-mediated necrotizing myopathy, inclusion body myositis, polymyositis and overlap myositis (Lundberg et al., 2021). According to its pathogenesis, B-cell depletion shows promise in controlling the progression of the disease. Four patients with antisynthetase syndrome treated with CAR-T have been reported and all have shown favorable outcomes (Müller et al., 2023; Taubmann et al., 2024). Three of them developed low-grade CRS, including one with ICANS, and symptoms improved with tolizumab treatment. Longer-term follow-up and additional clinical trials are needed to assess the longer-term therapeutic profile of CAR-T in antisynthetase syndrome.

### 4.5 CAR-T for ANCA-associated vasculitis

ANCA-associated vasculitis (AAV) encompasses a group of systemic autoimmune disorders marked by inflammation of small blood vessels and endothelial dysfunction. This condition can impact various organs and tissues, including the skin, lungs, kidneys, heart, gastrointestinal tract, and central nervous system. Based on clinical features and pathological manifestations, ANCA-associated vasculitis (AAV) can be categorized into three subgroups: granulomatosis with polyangiitis (GPA), microscopic polyangiitis (MPA), and eosinophilic granulomatosis with polyangiitis (EGPA) (Geetha and Jefferson, 2020). The pathogenesis of ANCA-associated vasculitis (AAV) remains not fully elucidated. However, Lodka et al. have demonstrated the feasibility and efficacy of CD19 CAR-T cell therapy for AAV treatment using a mouse model of MPO-AAV. Although myeloperoxidase (MPO)-ANCA was not completely eliminated, its levels were significantly reduced and demonstrated a strong protective effect against autoantibody-induced glomerulonephritis (Lodka et al., 2024). The application of this therapy for ANCA-associated vasculitis is currently restricted to vivo studies, and further data are needed to confirm its safety and efficacy.

### 4.6 CAR-T for rheumatoid arthritis

Rheumatoid arthritis (RA) is a chronic, inflammatory, systemic autoimmune disease that affects the joints with severity that varies from patient to patient (Cush, 2022), with risk factors including age, gender, genetics, and environmental exposures (smoking, air pollutants, and occupation).

RA is presently incurable, and the goal of treatment is to reduce pain and stop or slow further damage. The pathogenesis and etiology of rheumatoid arthritis (RA) remain largely unknown. However, protein citrullination has long been identified as a key feature that triggers the immune response associated with this condition. The presence of anti-citrullinated protein antibodies (ACPAs) in serum is one of the most specific serological markers for rheumatoid arthritis (RA) and is closely linked to the disease’s development (Curran et al., 2020; Scherer et al., 2020). Previous studies have shown that RA patients are significantly heterogeneous and different patients can exhibit different types of autoantibodies. Zhang et al. demonstrated that anti-FITC CAR-Ts can be specifically targeted to tumor cells or autoreactive B cells of RA patients with high levels of autoantibodies by citrullinated peptide epitopes labeled with FITC (fluorescein isothiocyanate), and that this approach can be used according to the individual characteristics of a given RA patient to eliminate the respective specific subpopulation of autoreactive cells. This opens up a new field of targeted therapy for autoimmune diseases (Zhang et al., 2021). However, the main limitation of this study is that it only demonstrated that it is possible to eliminate auto-reactive B cells *in vitro* and lacks evidence of therapeutic efficacy *in vivo*. Therefore, its feasibility and safety *in vivo* are unknown and need to be further explored in a large number of experiments.

In addition, citrullinated waveform protein (CV) has been identified as a specific antigen found exclusively in the extracellular matrix of inflamed synovial tissues in RA patients and has been suggested as a potential target for CAR-T cell therapy (Van Steendam et al., 2010). Preliminary data suggest that CAR-Tregs targeting citrullinated waveform protein (CV) may play a role in RA, but preclinical manifestations are not yet clear (Orvain et al., 2021).

## 5 Current clinical trials

A search of clinicaltrials.gov was performed for currently ongoing clinical trials of CAR-T for the treatment of SLE, SS, SSc, AAV, and RA. (Search date 2024.7.15, with no results found for ASS) (Table 2).

**TABLE 2:** Ongoing Clinical trials utilizing CAR-T to treat rheumatic immune diseases.

	Treatment	Target	Phase	Country
SLE	Biological: CD19 Universal CAR-γδ T Cells	CD19	Phase 1/2	China
	Biological: BRL-301	CD19	Not Applicable	China
	Drug: anti-CD19-CAR-T cells	CD 19	Phase 1	China
	Biological: anti-CD19-CAR-T cells	CD19	Early Phase 1	China
	Biological: anti-CD19-CAR-T cells	CD19	Phase 1	China
	Drug: CC-97540	CD19	Phase 1	US
	Drug: YTB323	CD19	Phase 1/2	China
	Biological: CABA-201	CD19	Phase 1/2	US
	Drug: CNCT19 cell injection	CD19	Early Phase 1	China
	Biological: Obecabtagene autoleucel (obe-cel)	CD19	Phase 1	UK
	Drug: RD06-04 Cells injection	CD 19	Not Applicable	China
	Biological: SC291	CD19	Phase 1	US
	Drug: Relma-cel	CD19	Phase 1	China
	Biological: Relma-cel	CD19	Phase 1	China
	Biological: single dose of CNCT19	CD19	Early Phase 1	China
	Drug: ATA3219	CD19	Phase 1	US
	Drug: anti-CD19 CAR T cell therapy	CD19	Phase 1/2	Germany
	Biological: CD19 CAR-T cell infusion	CD19	Phase 1/2	China
	Other: CAR T-cell therapy	CD 19	Phase 1	Thailand
	Biological: RJMty19 (CD19-CAR-DNT cells)	CD 19	Phase 1	China
	Biological: MB-CART19.1	CD19	Phase 1/2	Germany
	Biological: KYV-101 anti-CD19 CAR-T cell therapy	CD19	Phase 1/2	Germany
	Biological: KYV-101 anti-CD19 CAR-T cell therapy	CD19	Phase 1/2	US
	Biological: IM19 CAR-T cells	CD19	Not Applicable	China
	Biological: SCRI-CAR19v3	CD19	Phase 1	US
	Biological: T cell injection targeting CD19 chimeric antigen receptor	CD19	Not Applicable	China
	Drug: ADI-001	CD20	Phase 1	US
	Drug: Descartes-08	BCMA	Phase 2	US
	Drug: PRG-1801 (CAR-T against BCMA)	BCMA	Not Applicable	China
	Biological: LMY-920	BAFF	Phase 1	US
	Biological: IMPT-514	CD19 and CD20	Phase 1/2	US
	Drug: IMPT-514 CART Cell Injection	CD19/20	Early Phase 1	China
	Biological: Assigned Interventions CD19/BCMA CAR T-cells	CD19-BCMA	Early Phase 1	China
	Biological: CD19^−^ BCMA CAR-T cells	CD19^−^ BCMA	Phase 1/2	China
	Drug: GC012F injection	CD19-BCMA	Early Phase 1	China
	Drug: GC012F injection	CD19-BCMA	Phase 1	China
	Biological: PRG-2311	CD19-BCMA	Early Phase 1	China
	Biological: CD20/BCMA-directed CAR-T cells	CD20/BCMA	Phase 1	China
	Drug: FKC288	BCMA/CD19	Phase 1	China
	Biological: BCMA-CD19 cCAR T cells	BCMA-CD19	Phase 1	China
	Biological: BCMA/CD19 CAR-T cells	BCMA/CD19	Phase 1/2	China
	Biological: ATHENA CAR-T	ATHENA	Phase 1	China
	Biological: Anti-CD19^−^CD3E-CAR-T cells	CD19^−^CD3E	Not Applicable	China
SS	Biological: BRL-301	CD19	Not Applicable	Spain
	Biological: CD19 targeted CAR-T cells	CD19	Phase 1	China
	Biological: Anti-CD19^−^CD3E-CAR-T cells	CD19	Not Applicable	China
	Biological: T cell injection targeting CD19 chimeric antigen receptor	CD9	Early Phase 1	China
	Biological: T cell injection targeting CD19 chimeric antigen receptor	CD19	Not Applicable	China
	Biological: BCMA/CD19 CAR-T cells	BCMA/CD19	Phase 1/2	China
	Biological: CD19^−^ BCMA CAR-T cells	CD19^−^ BCMA	Phase 1/2	China
	Biological: Assigned Interventions CD19/BCMA CAR T-cells	CD19-BCMA	Early Phase 1	China
SSc	Drug: CC-97540	CD19	Phase 1	UK
	Biological: CD19 targeted CAR-T cells	CD19	Phase 1	China
	Biological: anti-CD19-CAR-T cells	CD19	Early Phase 1	China
	Drug: KYV-101	CD19	Phase 1	US
	Biological: KYV-101	CD19	Phase 1/2	US
	Biological: CABA-201	CD19	Phase 1/2	US
	Biological: BRL-301	CD19	Not Applicable	France
	Biological: Relma-cel	CD19	Phase 1	China
	Drug: anti-CD19 CAR T cell therapy	CD19	Phase 1/2	Germany
	Biological: Anti-CD19^−^CD3E-CAR-T cells	CD19	Not Applicable	China
	Biological: T cell injection targeting CD19 chimeric antigen receptor	CD19	Not Applicable	China
	Biological: T cell injection targeting CD19 chimeric antigen receptor	CD19	Early Phase 1	China
	Biological: CD19^−^ BCMA CAR-T cells	CD19^−^ BCMA	Phase 1/2	China
	Biological: Assigned Interventions CD19/BCMA CAR T-cells	CD19-BCMA	Early Phase 1	US
	Biological: CD20/BCMA-directed CAR-T cells	CD20/BCMA	Phase 1	China
AAV	Biological: CD19 targeted CAR-T cells	CD19	Phase 1	China
	Biological: Anti-CD19^−^CD3E-CAR-T cells	CD19	Not Applicable	China
	Biological: anti-CD19-CAR-T cells	CD19	Early Phase 1	China
	Biological: BRL-301	CD19	Not Applicable	China
	Biological: CD19^−^ BCMA CAR-T cells	CD19^−^ BCMA	Phase 1/2	China
RA	Biological: CD19 targeted CAR-T cells	CD19	Phase 1	China
	Biological: Anti-CD19^−^CD3E-CAR-T cells	CD19	Not Applicable	China
	Drug: KYV101	CD19	Phase 1/2	Germany
	Biological: anti-CD19-CAR-T cells	CD19	Early Phase 1	China
	Biological: BRL-301	CD19	Not Applicable	China
	Biological: T cell injection targeting CD19 chimeric antigen receptor	CD19	Not Applicable	China
	Biological: T cell injection targeting CD19 chimeric antigen receptor	CD19	Early Phase 1	China
	Biological: CD19^−^ BCMA CAR-T cells	CD19^−^ BCMA	Phase 1/2	China
	Biological: BCMA/CD19 CAR-T cells	CD19^−^ BCMA	Phase 1/2	China
	Biological: Assigned Interventions CD19/BCMA CAR T-cells	CD19^−^ BCMA	Early Phase 1	China

## 6 Challenges and strategies for CAR-T treatment of autoimmune diseases

**FIGURE 2:**
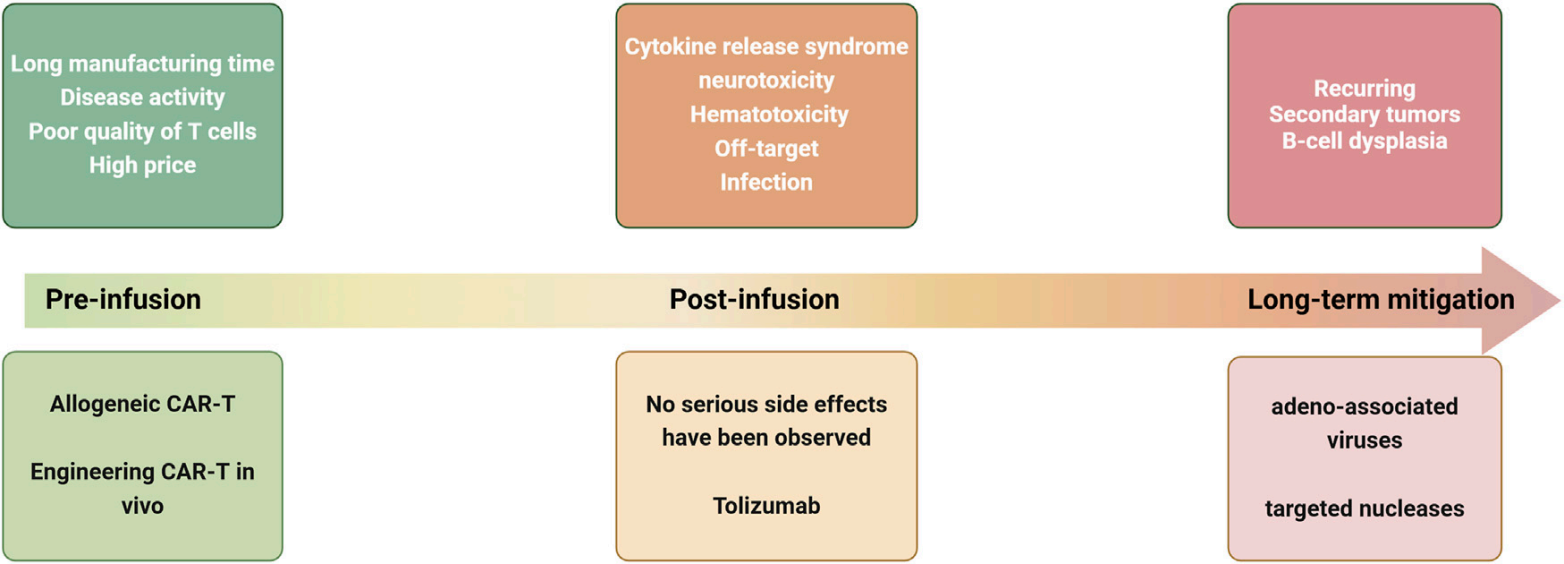
Current challenges and strategies for CAR-T treatment of autoimmune diseases at various stages of treatment.

While CAR-T therapy has demonstrated significant efficacy in treating both hematologic and solid tumors, it continues to encounter several challenges (Figure 2).

### 6.1 Pre-infusion

#### 6.1.1 CAR-T manufacturing is time-consuming

Autologous CAR-T cell therapy involves a highly complex process of customizing CAR-T cells following the isolation of each patient’s white blood cells. This procedure typically requires approximately 3 weeks to complete (Iyer et al., 2018; Köhl et al., 2018). In response to the problem of long *in vitro* manufacturing time, universal CAR-T (UCAR-T) has been proposed, which is taken from T cells of healthy donors, exempted from the action of heterologous reactive T cells by knocking out genes such as TCR, CD52, CD7, etc., and can be infused back in the spot without waiting, and the large-scale production can significantly reduce the price, which greatly improves the accessibility of CAR-T products (Lin et al., 2021), and this approach is expected to break the dilemma.

#### 6.1.2 Disease activity

During the time that autologous CAR-T is being manufactured *in vitro*, a number of patients will experience disease activity as a result of discontinuation of the drug and thus become ineligible for treatment. It may be possible to continue maintenance therapy with low-dose drugs during this period, and in addition, cyclophosphamide and fudarabine, which are used to clear lymphocytes, may have some therapeutic effect [(Green and Schultz, 2023; Srivastava et al., 2021)].

#### 6.1.3 Poor quality of T cells

Immune cells from healthy donors are usually unaffected by cancer effects or exposure to drugs, whereas in tumor patients, which may be related to immunosuppression, the tumor microenvironment, or having undergone multiple lines of therapy, T-cell dysfunction has been observed in tumor patients in the past, and these aberrant T-cells may affect the efficacy of CAR-T therapy (Park et al., 2018; Thommen and Schumacher, 2018). Given that patients with autoimmune diseases are frequently treated with glucocorticoids and other medications such as mycophenolate mofetil or cyclophosphamide, which can impact the quality and quantity of T cells, it is crucial to consider these factors when evaluating CAR-T therapy (Schulze-Koops, 2004). In a clinical study involving CAR-T therapy for five patients with severe, treatment-resistant systemic lupus erythematosus, stable and reproducible production of CD19CAR-T cells from peripheral blood was successfully achieved. This outcome occurred despite the patients having been pre-treated with T-cell-targeting medications, including mycophenolate mofetil and glucocorticoids, as part of their disease management (Mackensen et al., 2022).

#### 6.1.4 High price

The high cost of CAR-T therapy is a significant barrier for many patients (de Lima Lopes and Nahas, 2018), largely due to the complexity of the manufacturing process, which involves genetically modifying T cells, tailoring the treatment to each patient, implementing stringent quality-control measures, and requiring specialized facilities for cell production and processing (Levine et al., 2017; Choi et al., 2022). Back in 2017, there were two approved CAR-T drugs available in the United States: Kymriah and Yescarta. Kymriah has a listed price of US$475,000 and Yescarta has a listed price of US$373,000, and that doesn't even include the cost of possible side effects of the treatment, which is why it's currently only used to treat patients with particularly severe autoimmune diseases. Ideally, however, if long-term drug-free remission can be achieved with CAR-T therapy, the cost may be balanced against the cost of long-term repeated use of hormones, immunosuppressants, and biologics to control the disease. In addition to UCAR-T mentioned above, *in situ* cellular reprogramming, a potentially groundbreaking technology, also opens up possibilities to reduce costs and simplify the acquisition process (Parayath and Stephan, 2021). It targets and reprograms T cells *in vivo* to express CARs without the need for any *in vitro* manufacturing, thus greatly reducing costs, simplifying the acquisition process, and increasing availability. T-cell-targeted mRNA lipid nanoparticles (LNPs) have recently been found to deliver *in vivo* coding in mice, allowing the generation of functional CAR-T cells *in vivo*.

Rurik et al. (2022). This study demonstrated that a single injection of an off-the-shelf product can effectively reprogram the immune system, a technology that takes CAR-T cell therapy another step toward reducing costs and increasing accessibility.

### 6.2 Post-infusion

#### 6.2.1 Cytokine release syndrome and neurotoxicity

Although autoimmune diseases can be severe enough to cause death, the mortality rate is significantly lower than that of patients with malignant hematologic tumors. Therefore, the safety of the new treatment modality is the most concerned point for patients and doctors besides the efficacy. After the administration of CAR-T cells, their recognition of target cells triggers activation and proliferation, leading to the destruction of these target cells and the release of various cytokines, including IL-1, IL-2, IL-6, IL-8, and IL-10. The released cytokines further activate the patient’s immune cells, including T cells, B cells, monocytes, and macrophages, as well as non-immune cells such as endothelial cells. This cascade results in the release of additional cytokines, establishing a positive feedback loop known as cytokine release syndrome (CRS), which can escalate into a systemic inflammatory response with the potential to cause significant tissue and organ damage. Experiments have demonstrated that IL-6 plays a pivotal role in mediating cytokine release syndrome (CRS) (Morris et al., 2022; Garcia et al., 2021; Porter et al., 2011). The incidence of CRS is notably high in clinical trials targeting hematologic malignancies (Bouchkouj et al., 2019; Leahy et al., 2018). However, in CAR-T cell therapy trials for autoimmune diseases, no reports of grade 3 or higher CRS have been observed (Table 3). The good tolerability of CAR-T cell therapy in autoimmune diseases may be due to the fact that the amount of b-cells in patients with autoimmune diseases is many times lower than in patients with tumors. Therefore, cells carrying the target antigen (CD19) are quickly removed.

**TABLE 3:** CAR-T cell therapy for autoimmune disease cases with published results.

Disease	Age (years)	Sex (famale/male)	Remission (yes/no)	CRS	ICANS	Note	B cell aplasia	Time of onset of B cell proliferation	Source
SLE	20	Female	+	-	-		-	NA	Subklewe et al. (1990)
	22	Female	+	-	-		-	NA	Subklewe et al. (1990)
	23	Male	+	Grade 1	-		-	NA	Subklewe et al. (1990)
	24	Female	+	Grade 1	-	Better with tolizumab	-	NA	Subklewe et al. (1990)
	18	Female	+	-	-		-	NA	Subklewe et al. (1990)
	38	Female	+	-	-		-	NA	Subklewe et al. (1990)
	33	Female	+	-	-		-	NA	Subklewe et al. (1990)
	35	Female	+	-	-		-	NA	Subklewe et al. (1990)
	38	Female	+	Grade 1	-		-	3 m	Li et al. (2024)
	15	Female	+	-	-		+	NA	Krickau et al. (2024)
	29	Female	+	-	-		-	2 m	Wang et al. (2024b)
	46	Female	+	-	-		-	1 m	Wang et al. (2024b)
	32	Female	+	-	-		-	2 m	Wang et al. (2024b)
	36	Male	+	-	-		-	1 m	Wang et al. (2024b)
	20	Female	+	-	-		-	2 m	Wang et al. (2024b)
	23	Male	+	-	-		-	1 m	Wang et al. (2024b)
	23	Female	+	-	-		-	2 m	Wang et al. (2024b)
	16	Male	+	-	-		-	2 m	Wang et al. (2024b)
	31	Female	+	-	-		-	1 m	Wang et al. (2024b)
	36	Female	+	-	-		-	1 m	Wang et al. (2024b)
	17	Female	+	-	-		-	1 m	Wang et al. (2024b)
SS	76	Female	+	Grade 2	Grade 1	Better with tolizumab	NA	NA	Sheng et al. (2023)
SSc	60	Male	+	Grade 1	-		-	NA	Müller et al. (2024)
	36	Male	+	Grade 1	-		-	NA	Müller et al. (2024)
	37	Female	+	Grade 1	-		-	NA	Müller et al. (2024)
	47	Male	+	-	-		-	NA	Müller et al. (2024)
	45	Male	+	-	-		-	2 m	Subklewe et al. (1990)
	56	Male	+	-	-		-	3 m	Subklewe et al. (1990)
IMM	41	Male	+	Grade 1	-	Better with tolizumab	-	NA	Pecher et al. (2023)
	43	Female	+	Grade 1	Grade 1	Better with tolizumab	-	NA	Taubmann et al. (2024)
	42	Male	+	Grade 2	-	Better with tolizumab	-	NA	Lundberg et al. (2023)
	25	Male	+	NA		NA	-	3 m	Qin et al. (2024)

Abbreviations SLE, systemic lupus erythematosus; SS, Sjögren’s syndrome; SSc, systemic sclerosis; ASS, antisynthetase syndrome; CRS, cytokine release syndrome; ICANS, Immune Effector Cell-Associated Neurotoxicity Syndrome; NA, not applicable; m, month.

Note: “B cell aplasia “represents poor proliferation of B cells after clearance, and “-” represents B cells that did not experience poor proliferation after being cleared.

Additionally, some patients with acute lymphoblastic leukemia experience severe neurotoxicity, referred to as immune effector cell-associated neurotoxicity syndrome (ICANS), following CAR-T cell therapy (Santomasso et al., 2018). However, in the limited number of patients with autoimmune diseases currently undergoing CAR-T cell therapy, we have observed a relatively favorable tolerability profile, with no serious adverse effects reported (Table 3). Although some patients experienced a mild cytokine storm, this was effectively managed with prompt symptomatic treatment and tolizumab administration.

#### 6.2.2 Hematotoxicity

Hematotoxicity, which includes manifestations such as anemia, leukopenia, and thrombocytopenia (Fried et al., 2019), may occur early due to lymphocyte depletion before CAR-T therapy. Its late onset, however, is often associated with the severity of cytokine release syndrome (CRS) during treatment. This phenomenon has been observed in the management of both oncological and autoimmune diseases (Schett et al., 2023). Therefore, we need to closely monitor the changes in blood routine during the treatment period, and promptly transfuse component blood when necessary.

#### 6.2.3 Off-target

Off-target effects, where CAR-T cells either cross-react with non-target molecules or target antigens expressed on normal cells, are more commonly observed in tumor therapies (Castellarin et al., 2020; Chohan et al., 2023). These phenomena have not been reported in clinical trials of CAR-T therapy for autoimmune diseases. However, careful monitoring is essential to detect their potential occurrence during treatment.

#### 6.2.4 Infection

Patients undergoing CAR-T cell therapy face an elevated risk of infection due to several factors, including immunodeficiency from their underlying disease, prior cytotoxic treatments, lymphodepleting therapy, potential complications such as CRS and ICANS, as well as neutropenia and hypogammaglobulinemia. Infectious complications, both early and late, are commonly observed following CAR-T cell administration in the treatment of hematologic malignancies (An et al., 2024). However, such complications have not been specifically reported in the context of autoimmune diseases. Evidence-based guidelines for anti-infective prophylaxis following CAR-T cell therapy are lacking, and routine use of fungal and bacterial prophylaxis is uncommon. However, it should be actively considered in patients with neutropenia.

### 6.3 Long-term mitigation

#### 6.3.1 Recurring

A critical feature of CAR-T therapy is its capacity to identify target antigens and exert cytotoxic effects. When B cells proliferate again it may lead to disease relapse. However, a recent study found that after CAR-T treatment for autoimmune disease, newly emerged B cells predominantly expressed IgM and IgD, while memory B cells and plasmablasts were nearly absent. IgD is often expressed in relatively naïve B cells, including naïve B cells (IgD + CD27^−^), unswitched memory B cells (IgD+CD27^+^), So the phenomenon may be related to inflammatory suppression, deep B-cell clearance and subsequent reconstitution. (Schett et al., 2023; Shah et al., 2024).

#### 6.3.2 Secondary tumors

CAR transgenes are integrated into the genome of T cells primarily through viruses, a process that may lead to tumors. Secondary malignancies have been reported after CAR T-cell therapy, including myelodysplastic syndromes, bladder cancer, or nonmelanoma skin cancers (Locke et al., 2019; Si Lim et al., 2021), therefore, there have been attempts to use vectors and gene editing techniques other than retroviruses and lentiviruses to create a safer product, with adeno-associated viruses and targeted nucleases offering promise (Moço et al., 2020).

#### 6.3.3 B-cell dysplasia

Current clinical trials using CAR-T therapies have focused on removing B-cell markers such as CD19 or BCMA, and prolonged low levels of B-cells are often observed after CAR-T treatment of blood disorders, leaving patients susceptible to symptoms such as infections (An et al., 2024), and targeting infections that occur as a result of this B-cell dysplasia can be achieved with intravenous immune globulin. However, this has not been observed in autoimmune diseases for the moment (Schett et al., 2023) (Table 3). This regeneration of B cells is also a major point of difference in comparison to CAR-T therapy for tumors, the mechanism of which is currently unclear. Possible reasons for this include the fact that, unlike cancer patients, patients with autoimmune diseases receive fewer cytotoxic drugs, so stem cells and immune cells may be less affected (Schett et al., 2023). This may also be related to the distinct cellular niches for memory.

CAR T cells. The location of memory CAR-T cells is importantly linked to their persistence and B-cell reset. The earlier appearance of B-cell proliferation in lupus patients may be due to the fact that CAR T cells are located in secondary lymphoid organs, whereas in leukaemia memory CAR T cells are located in the bone marrow (Baker and June 2022). However, the proliferation of B-cells is theoretically incompatible with the long-term efficacy of CAR-t therapy, so a large number of clinical trials are needed to assess the long-term efficacy of CAR-T in autoimmune diseases.

## 7 Conclusion

As mentioned above, CAR-T has been explored for the treatment of a variety of autoimmune diseases, with current clinical trials showing good efficacy and a fair safety profile compared to tumors. However, there are some challenges that we need to continue to work to overcome before, during and after its treatment.

## 8 Future prosepects

CAR-T therapy has shown remarkable success in the treatment of hematological diseases and solid tumors, and as an emerging therapy in autoimmune diseases, it has also demonstrated certain potential. However, the number of patients with autoimmune diseases who have received this therapy is not large enough, the follow-up period is short, and the relevant clinical data are still scarce. So the longer-term efficacy and safety are still unclear. We expect that it will become a mature therapy for autoimmune diseases in the future.

## 9 Method

We searched PubMed and Web of science for articles published from From 1 January 2010 to 30 September 2024, using the search terms ‘CAR T cell’ and ‘autoimmune disease’ or ‘Rheumatic Autoimmune Disease’. We also searched for articles linked to the terms ‘B cell depletion’. We restricted our search to articles published in English. We included clinical studies, mechanistic studies, case reports, and case series, as well as reviews on CAR T-cell therapy into our references. Articles related to autologous and allogenic stem cell transplantation in autoimmune diseases, were also included.
